# Liver Fatty Acid Composition and Inflammation in Mice Fed with High-Carbohydrate Diet or High-Fat Diet

**DOI:** 10.3390/nu8110682

**Published:** 2016-10-29

**Authors:** Lorena Gimenez da Silva-Santi, Marina Masetto Antunes, Silvana Martins Caparroz-Assef, Fabiana Carbonera, Laureane Nunes Masi, Rui Curi, Jesuí Vergílio Visentainer, Roberto Barbosa Bazotte

**Affiliations:** 1 Department of Pharmacology and Therapeutics, State University of Maringá, Maringá 87020-900, Paraná, Brazil; lorenajufem@gmail.com (L.G.d.S.-S); marinaantunes_1992@hotmail.com (M.M.A.); smcassef@uem.br (S.M.C.-A.); 2 Department of Chemistry, State University of Maringá, Maringá 87020-900, Paraná, Brazil; fabianacarbonera@gmail.com (F.C.); jesuiv@gmail.com (J.V.V.); 3 Department of Physiology and Biophysics, Institute of Biomedical Sciences, University of São Paulo, São Paulo 05508-900, Brazil; laure_masi@hotmail.com (L.N.M.); curirui@gmail.com (R.C.)

**Keywords:** saturated fatty acids, monounsaturated fatty acids, polyunsaturated fatty acids, *n*-6/*n*-3 fatty acid ratio, desaturases, elongases, nitric oxide, myeloperoxidase, pro-inflammatory cytokines

## Abstract

Both high-carbohydrate diet (HCD) and high-fat diet (HFD) modulate liver fat accumulation and inflammation, however, there is a lack of data on the potential contribution of carbohydrates and lipids separately. For this reason, the changes in liver fatty acid (FA) composition in male Swiss mice fed with HCD or HFD were compared, at the time points 0 (before starting the diets), and after 7, 14, 28 or 56 days. Activities of stearoyl-CoA desaturase-1 (SCD-1), ∆-6 desaturase (D6D), elongases and de novo lipogenesis (DNL) were estimated. Liver mRNA expression of acetyl-CoA carboxylase 1 (ACC1) was evaluated as an additional indicator of the de novo lipogenesis. Myeloperoxidase activity, nitric oxide (NO) production, and mRNA expressions of F4/80, type I collagen, interleukin (IL)-6, IL-1β, IL-10, and tumor necrosis factor-α (TNF-α) were measured as indication of the liver inflammatory state. The HCD group had more intense lipid deposition, particularly of saturated fatty acids (SFAs) and monounsaturated fatty acids (MUFAs). This group also showed higher DNL, SCD-1, and D6D activities associated with increased NO concentration, as well as myeloperoxidase activity. Livers from the HFD group showed higher elongase activity, stored more polyunsaturated fatty acids (PUFAs) and had a lower omega-6/omega-3 fatty acid (*n*-6/*n*-3) ratio. In conclusion, liver lipid accumulation, fatty acids (FA) composition and inflammation were modulated by the dietary composition of lipids and carbohydrates. The HCD group had more potent lipogenic and inflammatory effects in comparison with HFD.

## 1. Introduction

Non-alcoholic fatty liver disease (NAFLD) is a condition whereby there is high hepatic lipid accumulation even under low alcohol intake [1]. NAFLD is not necessarily a disease, since it may be reversed by physical exercise [2], food restriction and body weight reduction [3]. However, some patients with NAFLD progress to nonalcoholic steatohepatitis (NASH) that can lead to fibrosis, cirrhosis, and eventually, liver failure [4].

NAFLD is the most common cause of liver diseases since this organ has a limited capacity for lipid storage. It is associated with obesity, insulin resistance, type 2 diabetes, hypertriglyceridemia, plasma non-high density lipoprotein cholesterol (HDL) high levels and cardiovascular disease [5,6,7]. The disease affects about 1 billion individuals worldwide [1].

In spite the fact that liver fat accumulation and liver inflammation are the main characteristics of NAFLD [8], the contribution of specific fatty acids (FA) toward the inflammatory process is not fully known. The change induced by high-fat diet (HFD) or high-carbohydrate diet (HCD) on liver fatty acid (FA) composition has not yet been compared.

We compared herein the changes in the liver FA composition in mice fed with HCD or HFD for 7, 14, 28 or 56 days as compared to the values before starting the diets (day 0). Activities of stearoyl-CoA desaturase-1 (SCD-1), ∆-6 desaturase (D6D), elongases and de novo lipogenesis (DNL) were estimated and gene expression of acetyl-CoA carboxylase 1 was evaluated. Nitric oxide content, myeloperoxidase activity and inflammatory gene expressions were measured as indication of the liver inflammatory state that is closely associated with fat deposition [4]. Mice fed with obesogenic diets are a well-established experimental model of liver steatosis [9,10] and inflammation [11].

## 2. Materials and Methods

### 2.1. Animals and Treatments

Male Swiss mice (*Mus musculus*), weighing about 35 g (six weeks of age), were obtained from the State University of Maringá Breeding Center and used in the experiments. Swiss mice are resistant to development of diet-induced obesity [12]. This mouse strain allowed us to compare the changes induced by either HCD or HFD without the jeopardizing effect of obesity. In accordance, there were no differences in initial and final body weights between the HCD and HFD groups.

Mice were maintained at 23 °C, with an automatically controlled photoperiod (12 h light/12 h darkness) and had free access to water and food.

The experimental protocol used (protocol 002/2014) was approved by the Animal Ethics Committee of The State University of Maringá (CEUA).

Mice were fed with standard rodent chow (Nuvilab^®^, Curitiba, PR, Brazil) before the initiation of the experimental protocol.

After three days of acclimatization, the animals were randomly divided into two groups and were allocated one per cage. One group was fed HFD and the other, HCD.

Amounts (g/100 g) of protein, carbohydrate and total fat in the HCD were 14.2, 73.8 and 4, respectively. Quantities (g/100 g) of protein, carbohydrate and total fat in the HFD were 20.3, 36.5 and 35.2, respectively.

The diets were prepared with highly refined ingredients purchased from Rhoster Company (Araçoiaba da Serra, SP, Brazil) and lard was used as a major source of fat. The HCD composition was based on purified diets for maintenance of laboratory adult rodents proposed by the American Institute of Nutrition (AIN-93-M) [13]. This latter diet is usually used as control. However, due to the high amount of carbohydrate, i.e., 73.8%, we herein named it a high carbohydrate diet.

Mice (total = 91) were fed with a HFD or HCD for 0, 7, 14, 28 or 56 days. After receiving HCD or HFD, mice fasted from 17:00 to 08:00 h, were killed by decapitation (to avoid any interference of anesthesia) and the livers removed and stored in liquid nitrogen. The inflammatory state of the liver was evaluated only in animals fed with HCD (*n* = 5) or HFD (*n* = 6) for 56 days.

FA composition of the diets was measured (Table 1). The main saturated fatty acid (SFA), monounsaturated fatty acid (MUFA) and polyunsaturated fatty acid (PUFA) in both HCD and HFD were: palmitic acid, oleic acid and linoleic acid, respectively. The diets had about the same omega-6/omega-3 fatty acid (*n*-6/*n*-3) ratio, i.e., 15/1.

### 2.2. Measurements of Diet and Liver Fatty Acid Composition

Total lipid contents of diets and livers were extracted using the method of Bligh and Dyer [14], in reduced scale.

The homogenized sample was weighed (1.000 ± 0.001 g) in 10 mL glass tubes and extraction performed by adding 2 mL methanol, 2 mL chloroform and 1 mL distilled water in different steps. The tubes were vortexed for 9 min and, after extraction, centrifuged at 3000 rpm for 5 min. The residual chloroform phase, containing total lipid extracted, was separated and the solvent removed using nitrogen gas flow. Fatty acid methyl esters (FAME) of diet and liver homogenates were prepared by ultrasound assisted total lipid methylation as described by Santos et al. [15]. FAME separation was performed by gas chromatography in a Thermo Scientific™ TRACE™ Ultra Gas Chromatographer (Thermo Scientific™, Waltham, MA, USA), fitted with a flame ionization detector (FID) and a fused-silica capillary column (100 m × 0.25 mm i.d., 0.25 µm cyanopropyl CP-7420 select FAME). The ultra-pure gas flows were 1.2 mL∙min^−1^ carrier gas (hydrogen), 30 mL∙min^−1^ make-up gas (nitrogen), 350 mL∙min^−1^ synthetic air and 35 mL∙min^−1^ hydrogen flame gas. The injected sample volume was 2.0 µL with split injection ratio 1:80. The injector and detector temperatures were 200 °C and 240 °C, respectively. The column temperature was maintained at 165 °C for 7 min, followed by a heating rate of 4 °C per min until reaching 185 °C, which was maintained for 4.67 min. After that, a new heating rate of 6 °C per min was applied until reaching 235 °C, which was maintained for 5 min, totalling 30 min of analysis.

Retention times and peak areas were determined using the Chrom-Quest™ software (Thermo Scientific™). For identification of FA, retention times were compared to those of standard methyl esters. FA contents in the diets and livers were expressed as mg/100 mg sample. 

### 2.3. Estimation of Enzyme Activities (SCD-1, D6D, Elongase) and DNL in the Liver

Enzyme activities and DNL were estimated as the product/precursor ratio of individual FA as follows: SCD-1 activity as the ratios of 16:1*n*-7/16:0 and 18:1*n*-9/18:0; D6D as the ratio of 18:3*n*-6/18:*2n*-6; elongase as the ratio of 18:0/16:0; and DNL as the ratio of 16:0/18:2*n*-6.

### 2.4. Determination of Nitric Oxide (NO) Production in the Liver

NO gas released from cells into the media reacts with water to produce nitrite and nitrate [16]. Nitrite and nitrate were then measured in the cell culture supernatant (200 μL) in triplicate. Griess reagent, containing sulfanilamide (1 g) in phosphoric acid (2.5 mL) and dihydrochloride of *N*-(1-naphtyl) ethylenediamine in milli-Q water (0.1 g), was added at room temperature. Absorbance was measured at 550 nm using an ELISA plate reader. NO production was calculated using a standard curve of sodium nitrite and results expressed as μM.

### 2.5. Determination of Myeloperoxidase Activity in the Liver

The livers were homogenized in phosphate buffered saline (PBS) and the homogenate was stirred in a vortex and centrifuged (2500 rpm) for 5 min.

The supernatant (10 μL) was added to each well in triplicate. PBS (0.2 mL), containing *o*-dianisidine dihydrochloride (4.2 mg), double-distilled water (22.5 mL), potassium phosphate buffer (2.5 mL, pH = 6), and H_2_O_2_ (10 μL, 1%) was also added. The enzyme reaction was stopped by addition of 30 μL sodium acetate (2.23 g in 20 mL of double-distilled water).

Myeloperoxidase activity was determined at 460 nm using a microplate spectrophotometer (Asys Expert Plus—Biochrom, Cambridge, UK).

### 2.6. Expressions of the De Novo Lipogenesis Synthesis Enzyme Acetyl-CoA Carboxylase 1 (ACC1) and Inflammatory Genes and Estimation of the Inflammatory Marker Index (IMI) in the Liver

Total RNA was extracted from 50 mg of liver using TRIzol reagent (Invitrogen Life Technologies, Waltham, MA, USA) and reverse transcribed to cDNA (High-Capacity cDNA kit, Applied Biosystems, Foster City, CA, USA). Gene expression was evaluated by real-time PCR using SYBR Green as fluorescent dye (Invitrogen Life Technologies). The primer sequences were: F4/80, NM_010130.4, sense CCTGAACATGCAACCTGCCAC, antisense GGGCAT GAGCAGBCTGTAGGATC, Type I collagen, NM_009931.2, sense CTCTATGTCCAAGGCAACGAG, antisense TCACAAACCGCACACCTG, IL-6, NM_001314054.1, sense, antisense, IL-1β, NM_008361.4, sense GGCAGCTACCTGTGTCTTTCCC, antisense ATATGGGTCCGACAGCACGAG, TNF-α, NM_001278601.1, sense TCTTCTCATTCCTGCTTGTGGC, antisense CACTTGGTGGTTTGCTACGAC G, IL-10, NM_010548.2, sense TGCCAAGCCTTATCGGAAATG, antisense AAATCGATGACAGCGCCTCAG, ACC1 NM_133360.2, sense GAGAGGGGTCAAGTCCTTCC, antisense AAAACATCCACTTCCACACACGA. The analysis of gene expression was carried out using a previously described method [17] with the 18S gene as internal control, NM_030720.1, sense CGCTACACTGACTGGCTCAG, antisense CAGGGACTTAATCAACGCAAG.

The IMI was calculated by the sum of expressions of pro inflammatory factors divided by the sum of expressions of anti-inflammatory factors as follows: interleukin (IL)-6 + IL-1β + tumor necrosis factor-α (TNF-α) + F4/80 + type 1 collagen/IL-10.

### 2.7. Statistical Analysis

Results are reported as means ± standard deviation of the means and were analyzed by one-way ANOVA. Tukey and Student *t*-test using Graph-Pad Prism Version 5.0 software (GraphPad Software, San Diego, CA, USA) were used to assess differences between means. *p*-values < 0.05 indicate statistical significance.

## 3. Results

### 3.1. Fatty Acid Composition of the Livers

In agreement with the diet composition (Table 1), livers from HCD and HFD mice had higher content of palmitic acid (SFA), oleic acid (MUFA) and linoleic acid (PUFA) as compared with other FAs (Table 2).

Lipid accumulation, calculated by the sum of all FA, was intensified (*p* < 0.05) during the experimental period for both HCD and HFD. The HCD group had high (*p* < 0.05) FA accumulation, particularly with respect to SFA and MUFA (Table 3). The HCD group exhibited low (*p* < 0.05) levels of PUFAs (*n*-3 and *n*-6), due to more intense reduction of linoleic acid, eicosapentaenoic acid (EPA) and docosahexaenoic acid (DHA) levels during the 56-day period (Table 2 and Table 3).

Arachidonic acid (AA) content was increased during the 56-day period in the livers of both groups but the changes were more pronounced in HFD mice (Table 2). Decrease of *n*-6/*n*-3 fatty acid ratios were observed in the HFD group throughout the 56-day period (Table 3). The PUFA/SFA ratio was reduced by both HCD and HFD, however, it was more pronounced in the HCD group (Table 3).

The MUFA/SFA ratio was increased in livers from the HCD group from day 7 *(*Table 3).

### 3.2. Estimated Activities of SCD-1, D6D, Elongase and DNL in the Liver

Elevations (*p* < 0.05) of SCD-1 and D6D activities during the 56-day period were observed in the livers of HCD mice (Table 4). Liver ACC1 expression was increased in the HCD mice; the values expressed as mean ± standard deviation of 5–6 mice per group were: 1.02 ± 0.22 for HCD and 0.634 ± 0.18 for HFD. These values were significantly different as indicated by the Student *t*-test for *p* < 0.05.

The elongase activities were increased (*p* < 0.05) and decreased (*p* < 0.05) in the liver of the HFD and the HCD groups, respectively, during the experimental period. DNL was increased throughout the study either by HCD or HFD but the changes were more pronounced in HCD mice.

### 3.3. Inflammatory Parameters

#### 3.3.1. Liver Myeloperoxidase Activity and Nitric Oxide Levels

Eleven mice that received the diets for 56 days were used in this analysis. Myeloperoxidase activity and NO levels were increased (*p* < 0.05) after 56 days of receiving HCD as compared to the HFD group (Table 5).

#### 3.3.2. mRNA Expressions of F4/80, Type I Collagen, IL-6, IL-1β, IL-10, and TNF-α in the Liver

Eleven mice that received the diets for 56 days were used in this analysis. F4/80, type I collagen, IL-6, IL-1β, TNF-α and IL-10 (Table 6) mRNA expressions were not significantly different between HCD and HFD.

However, the IMI was increased in the liver of HCD animals (*p* < 0.05) indicating a more intense inflammatory state in this group.

## 4. Discussion

### 4.1. Liver FA Accumulation

In agreement with previous studies in mice [12] and humans [18], livers from the HFD and HCD groups had higher content of palmitic acid, stearic acid, oleic acid, linoleic acid, and arachidonic acid in comparison with other FA (Table 2).

The more intense (*p* < 0.05) deposition of FA in the HCD group, which was inferred from the sum of all FA, was due to MUFAs being the main contributors (Table 3). These results may be explained as a consequence of increased DNL [19] and SCD-1 activity (Table 4). In fact, increased carbohydrate supply has been reported to stimulate DNL and SCD-1 activity [20].

The mechanisms by which DNL increases due to high carbohydrate diet involve SREBP-1c and ChREBP, which influence the expression of key genes involved in DNL such as acetyl-CoA carboxylase. Acetyl-CoA, generated from glucose, activates the transcription factors SREBP1c and ChREBP in the liver, which stimulate DNL [21]. In contrast, dietary FA are directly incorporated into triglycerides by diacylglycerol acyltransferase, and are not able to activate DNL [22]. *n*-3 PUFAs, found in high concentrations in the liver of HFD mice, prevent liver steatosis by inhibiting DNL via down regulation of SREBP-1c gene expression and lipogenic gene (FAS, ACC, and SCD-1) expressions, and stimulation of FA oxidation [23].

PUFA levels are modulated by the ingestion of linoleic acid and α-linolenic acid [24]. In accordance with this statement, high (*p* < 0.05) hepatic levels of linoleic acid and α-linolenic acid and their products of elongation and desaturation were found in HFD mice (Table 2) as consequence of the high amount of essential PUFA in their diet (Table 1).

Low PUFA/SFA ratio has been associated with increased risk of atherosclerosis, cardiovascular diseases and diabetes [25]. Low liver PUFA/SFA ratio may indicate a predisposition for HCD mice to develop these latter diseases (Table 3).

In accordance with the fact that *n*-3 PUFAs reduce lipid content in NAFLD [26], the HFD group accumulated less FA, as expected by the high (*p* < 0.05) levels of EPA and DHA (Table 2). In addition, the serum levels of glucose, total cholesterol and triglyceride were not different between the two groups.

### 4.2. Activities of Elongase, Desaturase and SCD-1

Elongase is regulated by diet composition and changes in its activity can lead to alterations in cell lipid composition [27,28]. Moon et al. [29] have reported accumulation of palmitic acid, palmitoleic acid and reduced contents of stearic acid and oleic acid in knockout mice for this enzyme.

In rodents, about 90% of the stearic acid is synthesized from palmitic acid by elongase activity [29]. In accordance, mice fed with HFD, presenting high (*p* < 0.05) elongase activity, also had higher stearic acid levels (Table 2). The contents of vaccenic acid, an elongation product of palmitoleic acid [30], was higher (*p* < 0.05) in the HCD group, in spite of the fact that the HCD group had lower elongase activity (Table 4). These results may be explained by the higher availability of palmitoleic acid (a precursor of vaccenic acid) in the livers from the HCD group (Table 2).

SCD-1 introduces a double bond on the ∆9 position in palmitic acid and stearic acid whereas D6D causes an unsaturation in linoleic acid and α-linolenic acid. These enzymes are expressed in several tissues, including the liver [30], and have an important role not only in the maintenance of plasma membrane lipid composition but also in the production of lipid signaling molecules such as eicosanoids [31].

An increase in SCD-1 and D6D activity was observed in the livers of the HCD group (Table 4). The changes in SCD-1 may be attributed to the high carbohydrate content of the diet [32,33]. The higher SFA level in the liver from the HCD group (Table 3) also increases SCD-1 activity and its RNAm expression [34]. Knockout mice for hepatic SCD-1 were protected against liver steatosis induced by HCD and this was associated with low rates of DNL [35].

In humans, higher activity of SCD-1 and lower activity of elongase, as described herein for the HCD group, are associated with obesity [36], hypertriglyceridemia [37], metabolic syndrome [27,38], insulin resistance [39], and NASH [17].

In accordance with the fact that the HFD group had lower (*p* < 0.05) D6D and SCD-1 activity (Table 4), Vessby et al. [40] reported that this enzyme activity is inhibited by AA and PUFA which are increased in the HFD group (Table 2 and Table 3).

### 4.3. Inflammation Associated with Liver FA Accumulation

FA modulate eicosanoid metabolism, act on plasma membrane and cytosolic signaling processes and influence activities of transcription factors involved in inflammation like NFκB and PPAR-γ [41].

Liver content of AA, a precursor of pro-inflammatory prostaglandins, thromboxans, leukotrienes and lipoxins [42] was increased in the HFD group (Table 2).

MUFAs have anti-inflammatory effects [43], and were found at high levels in the liver from the HCD group. Thus, a more prominent liver pro-inflammatory state in the HFD group could be predicted. However, the HFD liver had higher (*p* < 0.05) levels of *n*-3 PUFAs (Table 2) that have anti-inflammatory effects [44,45]. This latter group had lower (*p* < 0.05) *n*-6/*n*-3 fatty acid ratio (Table 3) in spite of the fact that similar *n*-6:*n*-3 fatty acid proportions were described for both high carbohydrate diet and high fat diet. The HFD group also had lower SFA levels (Table 3), especially palmitic acid (Table 2), which is able to raise nuclear factor kappa B (NF-κB) activity by binding to Toll-like receptor-2 (TLR-2) and 4, increasing expressions of pro-inflammatory cytokines such IL-6 and TNF-α [18,46].

Considering the pro-inflammatory properties of AA and SFA, and the anti-inflammatory effects of *n*-3 PUFA, the inflammatory states of the livers from the HCD and HFD groups were compared.

The liver inflammatory state was herein evaluated by the measurements of myeloperoxidase activity, NO content and inflammatory gene expressions (F4/80, type I collagen, IL-6, IL-1β, TNF-α and IL-10).

Increased myeloperoxidase activity and NO contents in the liver indicate a more prominent (*p* < 0.05) inflammatory state in HCD mice (Table 5). Myeloperoxidase activity is a marker of tissue neutrophil infiltration [47]. The higher (*p* < 0.05) activity of myeloperoxidase (Table 5) in the livers of HCD mice suggests a more intense hepatic neutrophil infiltration [47].

Pro-inflammatory cytokines such as IL-1 β, TNF-α, IFN-γ [48] and nuclear factor kappa B [49] increase inducible nitric oxide synthase (iNOS) expression [48] and consequently NO production [18]. The increased (*p* < 0.05) NO production (Table 5) in HCD animals also supports a more intense inflammation state in the liver. In agreement with these findings, linoleic acid, α-linolenic acid, EPA and DHA, whose levels are increased (*p* < 0.05) in the liver from the HFD group (Table 2), decrease NF-κB DNA-binding activity [44,45]. Furthermore, palmitic acid content, which is increased (*p* < 0.05) in the livers of the HCD group (Table 2), enhances NF-κB activity [18,46,50].

The F4/80 is one of the most specific cell-surface markers for macrophages. F4/80 is highly and constitutively expressed in resident tissue macrophages, including Kupffer’s cells in the liver [51]. There were no differences in F4/80 mRNA expression, indicating similar macrophage infiltration in livers from both HCD and HFD groups (Table 6).

There were no differences in liver cytokine mRNA expression between the HCD and HFD groups. However, the IMI was increased in livers from the HCD group mainly because IL-10 was poorly expressed in this group (Table 6). This information, together with the results of myeloperoxidase, NO and high *n*-6/*n*-3 fatty acid ratio, indicates an increased inflammatory state in livers from mice fed with HCD for 56 days, as compared with the HFD group. In accordance with these results, high postprandial blood glucose levels induced by high carbohydrate food intake increase NO generation, that in turn can combine with superoxide to produce peroxynitrite, a potent long-lived pro-oxidant molecule, contributing either to acute or chronic low-grade inflammation [52].

**Figure 1:**
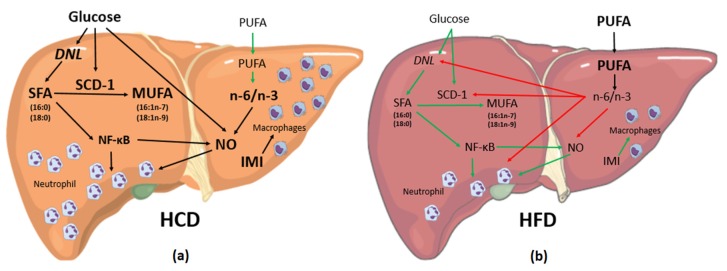
Summary of the results in livers from mice fed with either high carbohydrate diet (HCD) or high fat diet (HFD). Bold words indicate higher availability in comparison with the other group; black arrows indicate strong stimulation; green arrows indicate weak stimulation; red arrows represent inhibition. The HCD (**a**) generates glucose in excess that reaches the liver at high quantities and stimulates DNL and SCD-1 activity. This stimulation leads to production of high quantities of SFAs and MUFAs. SFA, especially 16:0, activates NF-κB that increases NO production and tissue neutrophil infiltration indirectly. In addition, low quantities of *n*-3 PUFA and increased *n*-6/*n*-3 PUFA ratio, prompt inflammation; HFD mice (**b**) had lower FA accumulation and lower *n*-6/*n*-3 PUFA ratio in the liver that is well known as an anti-inflammatory condition. Low *n*-6/*n*-3 PUFA ratio inhibits DNL, reduces SCD-1 and NF-κB activities and results in low lipid accumulation. Abbreviations: DNL, de novo lipogenesis; SCD-1, stearoyl CoA desaturase-1; SFA, saturated fatty acids; MUFAs, monounsaturated fatty acids; PUFAs, polyunsaturated fatty acids; NF-κB, nuclear factor kappa B; NO, nitric oxide; IMI: inflammatory marker index.

The results in livers from mice fed with HCD or HFD are summarized (Figure 1).

## 5. Conclusions

The proportion of fat and carbohydrates in the diet modulated the deposition of lipids and composition of liver fatty acids. The liver from the HFD group had higher elongase activity and stored more *n*-3 and *n*-6 PUFAs. Increased lipid deposition particularly of SFAs and MUFAs, higher SCD-1 and D6D activities and ACC1 expression and DNL were reported in the liver from the HCD group. These changes were associated with a more intense liver inflammation state (Figure 1).

## Figures and Tables

**Table 1:** Fatty acid composition (mg 100/g) of the high-carbohydrate (HCD) and the high-fat (HFD) diets.

Fatty Acids	Diets	
	HCD	HFD
Myristic Acid (14:0)	88.5 ± 0.9	386.9 ± 6.4 *
Palmitic Acid (16:0)	1141.8 ± 12.8	6530.4 ± 58.6 *
Stearic Acid (18:0)	555.0 ± 7.0	3169.1 ± 19.9 *
Hipogeic Acid (16:1*n*-9)	17.2 ± 0.4	104.1 ± 4.9 *
Palmitoleic Acid (16:1*n*-7)	87.7 ± 0.5	520.1 ± 6.3 *
Oleic Acid (18:1*n*-9)	1793.2 ± 12.1	10,678.8 ± 74.9 *
Vaccenic Acid (18:1*n*-7)	133.1 ± 1.5	811.15 ± 22.9 *
Linoleic Acid (18:2*n*-6)	1155.9 ± 9.7	6653.5 ± 38.7 *
α-Linolenic Acid (18:3*n*-3)	76.8 ± 0.5	403.5 ± 19.2 *
SFAs	1785.3 ± 14.6	10,086.5 ± 62.2 ^a^
MUFAs	2031.2 ± 12.2	12,114.2 ± 78.8 *
PUFAs	1232.8 ± 9.7	7057.0 ± 43.2 *
Total *n*-6 PUFA	1155.9 ± 9.7	6653.5 ± 38.7 *
Total *n*-3 PUFA	76.8 ± 0.5	403.47 ± 19.2 *

Results expressed as mean ± standard deviation of three replicates. Abbreviations: SFA: saturated fatty acids; MUFAs: monounsaturated fatty acids; PUFAs: polyunsaturated fatty acids; *n*-6: omega-6 fatty acids; *n*-3: omega-3 fatty acids. * *p* < 0.05 (HCD vs. HFD).

**Table 2:** Fatty acid composition (mg 100/g of sample) in the liver from mice fed with High-carbohydrate diet (HCD) or High-fat diet (HFD) diet at 0 (before starting the diets), or after 7, 14, 28 or 56 days.

Fatty Acids		0 (Day)	7 (Day)	14 (Day)	28 (Day)	56 (Day)
Myristic Acid (14:0)	HCD	26.4 ± 2.0	31.5 ± 1.1	38.8 ± 1.1 ^a^	26.6 ± 1.4 ^c^	58.6 ± 4.3 ^a,b,c,d^
	HFD		19.8 ± 1.4 ^a,^*	15.1 ± 0.9 ^a,b,^*	15.1 ± 0.7 ^a,b,^*	14.3 ± 0.9 ^a,b,^*
Palmitic Acid (16:0)	HCD	1084.9 ± 22	1387.5 ± 12.5 ^a^	1299.0 ± 23.8 ^a,b^	1063.3 ± 22.9 ^b,c^	1916.8 ± 29.6 ^a,b,c,d^
	HFD		1219.5 ± 10.4 ^a,^*	1154.1 ± 6.1 ^a,b,^*	1288.6 ± 19.9 ^a,b,c,^*	1314.7 ± 0.3 ^a,b,c,^*
Stearic Acid (18:0)	HCD	381.0 ± 28.3	415.3 ± 3.6	400.6 ± 23.2	311.9 ± 26.8 ^a,b,c^	415.4 ± 30.2 ^d^
	HFD		503.2 ± 5.5 ^a,^*	611.1 ± 8.2 ^a,b,^*	633.3 ± 39.3 ^a,b,^*	525.8 ± 13.1 ^a,c,d,^*
Heneicosanoic Acid (21:0)	HCD	27.3 ± 2.2	48.5 ± 2.1 ^a^	45.6 ± 2.8 ^a^	44.5 ± 4.3 ^a^	69.1 ± 1.6 ^a,b,c,d^
	HFD		32.4 ± 1.3 ^a,^*	33.0 ± 1.8 ^a,^*	44.5 ± 2.5 ^a,b,c^	61.5 ± 0.9 ^a,b,c,d^*
Tetracosanoic Acid (24:0)	HCD	26.2 ± 0.5	20.2 ± 0.2 ^a^	17.2 ± 1.2 ^a,b^	7.2 ± 0.3 ^a,b,c^	9.1 ± 0.9 ^a,b,c,d^
	HFD		26.8 ± 0.9 *	28.5 ± 2.0 *	24.2 ± 0.8 ^c,^*	20.9 ± 0.6 ^a,b,c,d,^*
7-hexadecanoic Acid (16:1*n*-9)	HCD	23.8 ± 0.4	29.8 ± 0.5 ^a^	39.7 ± 0.4 ^a,b^	34.0 ± 2.2 ^a,b,c^	64.1 ± 0.9 ^a,b,c,d^
	HFD		21.9 ± 0.6 *	18.1 ± 1.1 ^a,b,^*	17.1 ± 1.0 ^a,b,^*	32.3 ± 1.7 ^a,b,c,d,^*
Palmitoleic Acid (16:1*n*-7)	HCD	82.6 ± 3.0	191.0 ± 7.8 ^a^	250.3 ± 13.0 ^a,b^	209.1 ± 19.4 ^a^	524.3 ± 25.0 ^a,b,c,d^
	HFD		57.0 ± 3.7 ^a,^*	43.8 ± 0.8 ^a,b,^*	46.2 ± 1.4 ^a,b,^*	53.0 ± 2.3 ^a,c,d,^*
Oleic Acid (18:1*n*-9)	HCD	710.7 ± 46.4	1294.1 ± 16.9 ^a^	1613.4 ± 25.9 ^a,b^	1479.0 ± 104.8 ^a^	3077.9 ± 125.4 ^a,b,c,d^
	HFD		888.0 ± 7.8 ^a,^*	852.6 ± 20.9 ^a,^*	937.3 ± 41.9 ^a,^*	1152.7 ± 46.4 ^a,b,c,d,^*
Vaccenic Acid (18:1*n*-7)	HCD	76.6 ± 3.7	133.5 ± 4.4 ^a^	159.9 ± 3.1 ^a^	212.4 ± 19.2 ^a,b,c^	488.7 ± 13.4 ^a,b,c,d^
	HFD		88.2 ± 1.2 ^a,^*	87.7 ± 2.8 ^a,^*	97.6 ± 1.2 ^a,b,c,^*	114.0 ± 2.3 ^a,b,c,d,^*
Linoleic Acid (18:2*n*-6)	HCD	1472.8 ± 21.9	1125.3 ± 36.0 ^a^	1040.2 ± 12.9 ^a^	621.3 ± 52.7 ^a,b,c,d^	685.0 ± 24.3 ^a,b,c,d^
	HFD		1307.0 ± 19.0 ^a,^*	1274.9 ± 29.1 ^a,^*	1176.0 ± 37.7 ^a,b,c,^*	1066.1 ± 4.4 ^a,b,c,d,^*
γ-linolenic Acid (18:3*n*-6)	HCD	51.6 ± 0.6	48.6 ± 1.4	43.7 ± 2.9 ^a,b^	27.0 ± 1.7 ^a,b,c^	41.1 ± 1.4 ^a,b,d^
	HFD		36.3 ± 2.2 ^a,^*	40.0 ± 1.9 ^a^	25.0 ± 1.5 ^a,b,c^	20.3 ± 0.6 ^a,b,c,d,^*
Arachidonic Acid (20:4*n*-6)	HCD	453.4 ± 31.0	541.4 ± 10.9	508.6 ± 19.7	443.8 ± 44.2 ^b^	606.1 ± 40.7 ^a,d^
	HFD		585.5 ± 1.0 ^a,^*	679.7 ± 4.2 ^a,b,^*	778.0 ± 28.6 ^a,b,c,^*	700.5 ± 24.7 ^a,b,d,^*
Docosatetraenoic Acid (22:4*n*-6)	HCD	18.5 ± 1.5	20.7 ± 0.6	19.7 ± 1.3	10.9 ± 0.7 ^a,b,c^	16.1 ± 1.6 ^b,d^
	HFD		24.1 ± 0.6 ^a,^*	27.3 ± 1.7 ^a,b,^*	26.8 ± 0.5 ^a,^*	27.5 ± 1.1 ^a,b,^*
Docosapentaenoic Acid (22:5*n*-6)	HCD	17.8 ± 1.4	41.2 ± 1.1 ^a^	47.2 ± 4.5 ^a^	31.2 ± 3.0 ^a,b,c^	65.5 ± 4.4 ^a,b,c,d^
	HFD		29.0 ± 1.2 ^a,^*	37.7 ± 3.0 ^a,b^	31.6 ± 1.2 ^a,c^	30.5 ± 1.0 ^a,c,^*
α-Linolenic Acid (18:3*n*-3)	HCD	60.1 ± 4.4	29.5 ± 0.8 ^a^	24.2 ± 0.4 ^a^	7.9 ± 0.4 ^a,b,c^	9.8 ± 0.4 ^a,b,c^
	HFD		35.8 ± 0.9 ^a,^*	26.6 ± 0.6 ^a,b,^*	20.1 ± 0.9 ^a,b,c,^*	18.1 ± 0.2 ^a,b,c,^*
Eicosapentaenoic Acid (20:5*n*-3)	HCD	8.1 ± 0.5	6.5 ± 0.2 ^a^	5.0 ± 0.4 ^a,b^	4.1 ± 0.2 ^a,b^	3.5 ± 0.1 ^a,b,c^
	HFD		8.2 ± 0.2 *	9.2 ± 0.6 ^a,^*	7.1 ± 0.3 ^b,c,^*	6.7 ± 0.1 ^a,b,c,^*
Docosahexaenoic Acid (22:6*n*-3)	HCD	346.4 ± 25.7	394.9 ± 9.7 ^a^	281.1 ± 11.8 ^a,b^	213.4 ± 17.7 ^a,b^	244.7 ± 12.4 ^a,b,c^
	HFD		460.3 ± 7.6 ^a,^*	490.9 ± 9.9 ^a,^*	547.8 ± 24.7 ^a,b,c,^*	464.7 ± 15.3 ^a,d,^*

Results expressed as mean ± standard deviation of three replicates. HCD: High-carbohydrate diet; HFD: High fat diet. *p* < 0.05 as compared with day 0 ^a^, day 7 ^b^, day 14 ^c^ and day 28 ^d^, and HCD group *.

**Table 3:** Fatty acid family composition (mg 100/g of sample), and *n*-6/*n*-3 fatty acid, PUFA/SFA and MUFA/SFA ratios in the liver from mice fed with high carbohydrate diet (HCD) or high fat diet (HFD) at 0 (before starting the diets) or after 7, 14, 28 or 56 days.

Fatty Acids		0 (Day)	7 (Day)	14 (Day)	28 (Day)	56 (Day)
SFA	HCD	1545.9 ± 35.7	1903.0 ± 13.3 ^a^	1801.3 ± 33.4 ^a,b^	1453.6 ± 35.6 ^a,b,c^	2469.0 ± 42.5 ^a,b,c,d^
	HFD		1801.7 ± 12.0 ^a,^*	1841.9 ± 10.6 ^a,^*	2005.7 ± 44.1 ^a,b,c,^*	1937.2 ± 13.2 ^a,b,c,d,^*
MUFA	HCD	893.7 ± 46.6	1648.3 ± 19.1 ^a^	2063.4 ± 29.1 ^a,b^	1934.6 ± 108.3 ^a,b^	4155.0 ± 128.6 ^a,b,c,d^
	HFD		1055.0 ± 8.7 ^a,^*	1002.2 ± 21.1 *	1098.2 ± 42.0 ^a,^*	1351.9 ± 46.5 ^a,b,c,d,^*
PUFA	HCD	2428.8 ± 46.1	2208.2 ± 38.9 ^a^	1969.7 ± 26.9 ^a,b^	1359.7 ± 71.2 ^a,b,c^	1671.8 ± 49.2 ^a,b,c,d^
	HFD		2486.3 ± 20.7 *	2586.5 ± 31.3 ^a,b,^*	2612.4 ± 53.5 ^a,b,^*	2334.4 ± 29.4 ^a,b,c,d,^*
*n*-6	HCD	2014.2 ± 38.0	1777.3 ± 37.7 ^a^	1659.5 ± 24.2 ^a^	1134.2 ± 68.9 ^a,b,c^	1413.7 ± 47.6 ^a,b,c,d^
	HFD		1981.9 ± 19.2 *	2059.7 ± 29.6 ^b,^*	2037.5 ± 47.4 *	1844.9 ± 25.1 ^a,b,c,d,^*
*n*-3	HCD	414.6 ± 26.1	430.9 ± 9.7	310.2 ± 11.8 ^a,b^	225.5 ± 17.8 ^a,b,c^	258.0 ± 12.3 ^a,b,c^
	HFD		504.4 ± 7.7 ^a,^*	526.8 ± 10.0 ^a,^*	574.9 ± 24.7 ^a,b,c,^*	489.5 ± 15.3 ^a,d,^*
PUFA/SFA	HCD	1.6 ± 0.03	1.2 ± 0.02 ^a^	1.1 ± 0.02 ^a^	0.9 ± 0.06 ^a,b,c^	0.7 ± 0.03 ^a,b,c,d^
	HFD		1.4 ± 0.01 ^a,^*	1.4 ± 0.01 *	1.3 ± 0.03 ^a,b,c,^*	1.2 ± 0.01 ^a,b,c,d,^*
MUFA/SFA	HCD	0.6 ± 0.05	0.9 ± 0.02 ^a^	1.1 ± 0.02 ^a,b^	1.3 ± 0.06 ^a,b,c^	1.7 ± 0.04 ^a,b,c,d^
	HFD		0.6 ± 0.01 *	0.5 ± 0.02 *	0.5 ± 0.04 *	0.7 ± 0.04 ^a,b,c,d,^*
*n*-6/*n*-3	HCD	4.8 ± 0.07	4.1 ± 0.03 ^a^	5.3 ± 0.04 ^b^	5.0 ± 0.10 ^b^	5.5 ± 0.06 ^a,b^
	HFD		3.9 ± 0.02 ^a,^*	3.9 ± 0.02 ^a,^*	3.5 ± 0.05 ^a,^*	3.8 ± 0.03 ^a,^*
SUM	HCD	4868.4	5759.6	5834.3	4747.8	8295.8
	HFD		5343.0	5430.5	5716.3	5623.6

Results expressed as mean ± standard deviation of three replicates. Abbreviations: SFA: total saturated fatty acids; MUFAs: total monounsaturated fatty acids; PUFAs: total polyunsaturated fatty acids; SUM: sum of all fatty acids evaluated. HCD: High-carbohydrate diet; HFD: High fat diet. *p* < 0.05 as compared with 0 ^a^, day 7 ^b^, day 14 ^c^ and day 28 ^d^; and HCD group *.

**Table 4:** Estimations of enzyme activities (SCD-1, D6D and elongase) and of de novo lipogenesis (DNL) in the liver from mice fed with high carbohydrate diet (HCD) or high fat diet (HFD) at 0 (before starting the diets) or after 7, 14, 28 or 56 days.

		0 (Day)	7 (Day)	14 (Day)	28 (Day)	56 (Day)
**SCD-1 (16:1*n*-7/16:0)**	HCD	0.076 ± 0.003	0.138 ± 0.004 ^a^	0.193 ± 0.006 ^a,b^	0.197 ± 0.021 ^a,b^	0.274 ± 0.016 ^a,b,c,d^
	HFD		0.047 ± 0.003 ^a,^*	0.038 ± 0.001 ^a,b,^*	0.036 ± 0.0005 ^a,b,^*	0.040 ± 0.002 ^a,b,^*
**SCD-1 (18:1*n*-9/18:0)**	HCD	1.877 ± 0.252	3.116 ± 0.051 ^a^	4.036 ± 0.298 ^a^	4.786 ± 0.763 ^a,b^	7.449 ± 0.824 ^a,b,c,d^
	HFD		1.765 ± 0.015 *	1.395 ± 0.043 *	1.48 ± 0.159 *	2.195 ± 0.141 *
**D6D (18:3*n*-6/18:2*n*-6)**	HCD	0.035 ± 0.001	0.043 ± 0.0002 ^a^	0.042 ± 0.002 ^a^	0.044 ± 0.002 ^a^	0.060 ± 0.001 ^a,b,c,d^
	HFD		0.028 ± 0.001 ^a,^*	0.031 ± 0.001 ^a,b,^*	0.021 ± 0.0007 ^a,b,c,^*	0.019 ± 0.001 ^a,b,c,^*
**Elongase (18:0/16:0)**	HCD	0.352 ± 0.031	0.299 ± 0.002	0.309 ± 0.024	0.294 ± 0.031	0.217 ± 0.012 ^a,b,c,d^
	HFD		0.413 ± 0.001 ^a,^*	0.529 ± 0.007 ^a,b,^*	0.492 ± 0.031 ^a,b,^*	0.400 ± 0.010 ^c,d,^*
**DNL (16:0/18:2*n*-6)**	HCD	0.74 ± 0.06	1.23 ± 0.03 ^a^	1.25 ± 0.32 ^a^	1.72 ± 0.09 ^a,b,c^	2.80 ± 0.06 ^a,b,c,d^
	HFD		0.93 ± 0.02 ^a,^*	0.91 ± 0.02 ^a,^*	1.10 ± 0.05 ^a,b,c,^*	1.23 ± 0.03 ^a,b,c,d,^*

Results expressed as mean ± standard deviation. Abbreviations: SCD-1: stearoyl-CoA desaturase-1; D6D: ∆6-desaturase; DNL: de novo lipogenesis. HCD: High-carbohydrate diet; HFD: High fat diet. *p* < 0.05 as compared with day 0 ^a^, day 7 ^b^, day 14 ^c^ and day 28 ^d^, and HCD group *.

**Table 5:** Myeloperoxidase (MPO) activity and nitric oxide (NO) levels in the liver from mice fed either with high fat diet (HFD group) or with high carbohydrate diet (HCD group) for 56 days.

	HCD	HFD
MPO activity (DO 460 nm)	0.450 ± 0.106	0.302 ± 0.073 *
NO (Total Nitrite μM)	190.60 ± 54.55	112.11 ± 51.15 *

Results expressed as mean ± standard deviation. HCD: high-carbohydrate diet; HFD: high-fat diet. * *p* < 0.05 as compared with HCD group. HCD: High-carbohydrate diet; HFD: High fat diet. (*n* = 5–6 per group treated for 56 days).

**Table 6:** Expressions of inflammatory genes (by RT-PCR) in the liver from mice fed with either high fat diet (HFD group) or high carbohydrate diet (HCD group) for 56 days.

Gene	HCD	HFD
F4/80	1.02 ± 0.2	1.01 ± 0.4
Type I collagen	1.02 ± 0.2	0.87 ± 0.1
IL-6	1.24 ± 0.9	1.40 ± 0.7
IL-1β	1.10 ± 0.5	0.91 ± 0.3
TNF-α	1.20 ± 0.7	1.20 ± 0.6
IL-10	1.10 ± 0.6	1.80 ± 0.7
Inflammatory marker index (IMI)	25.06 ± 0.5	17.87 ± 0.5 *

mRNA gene expression in the liver. 18S was used as housekeeping gene. Results are expressed as means ± standard deviation of 5–6 mice per group. HCD: high-carbohydrate diet; HFD: high-fat diet; TNF: tumor necrosis factor; IL: interleukin; Inflammatory marker index = (IL-6 + IL-1β + TNFα + F4/80 + type 1 collagen)/IL-10. HCD: High-carbohydrate diet; HFD: High fat diet. * *p* < 0.05 as compared with the HCD group.
